# Incidence and predictors of neonatal hyperbilirubinemia requiring phototherapy: A prospective cohort study at a tertiary hospital in Uganda

**DOI:** 10.1371/journal.pone.0349861

**Published:** 2026-06-10

**Authors:** Hamdi Mohamed Yusuf, Munanura Turyasiima, Jolly Nankunda, Sabinah Wesigemukama Twesigemukama, Walufu Ivan Egesa, Abdullahi Mohamed Abdulle, Walyeldin Elfakey, Zakaria Abdi Said, Ahmed Hassan Mohamud, Joshua Epuitai, Feisal Dahir Kahie, Hanan Asad Hassan, Awil Abdulkadir Abdi, Theoneste Hakizimana, Abshir Mohamed Hirsi, Martin Nduwimana

**Affiliations:** 1 Department of Pediatrics and Child Health, Kampala International University, Kampala, Uganda; 2 Department of Standards Compliance, Accreditation and Patient Protection, Ministry of Health, Kampala, Uganda; 3 Mulago Specialized Women and Neonatal Hospital, Kampala, Uganda; 4 Department of Pediatrics, Nile International Hospital, Jinja, Uganda; 5 James Lind Institute, Geneva, Switzerland; 6 Department of Nursing, Faculty of Health Services, Busitema University, Mbale, Uganda; 7 Department of Internal Medicine, Kampala International University, Bushenyi, Uganda; 8 Department of Obstetrics and Gynecology, Kampala International University, Bushenyi, Uganda; Shoklo Malaria Research Unit, THAILAND

## Abstract

**Background:**

Neonatal hyperbilirubinemia remains a leading cause of preventable neurological impairment and death in low-resource settings, particularly in sub-Saharan Africa. Despite its burden, data on incidence and risk factors in Uganda are limited. This study aimed to determine the incidence and predictors of hyperbilirubinemia among neonates at a Ugandan referral hospital.

**Methods:**

A prospective cohort study was conducted at Mubende Regional Referral Hospital from November 2024 to February 2025. A total of 539 mother-infant pairs were enrolled, with 534 completing follow-up. Neonates were followed from birth up to 7 days of life. Data on maternal and neonatal characteristics were collected through interviews and medical records. Laboratory tests included total serum bilirubin and ABO/Rh blood grouping. Modified Poisson regression was used to identify independent predictors of hyperbilirubinemia, with statistical significance set at p < 0.05.

**Results:**

The risk of hyperbilirubinemia requiring phototherapy was increased by close to two times among neonates born to mothers who were **underweight, overweight, or obese (based on maternal BMI during pregnancy)** (aIRR = 1.703, CI = 1.082–2.680, P = 0.021; aIRR = 1.916, CI = 1.447–3.183, P < 0.001; aIRR = 1.930, CI = 1.233–3.022, P = 0.004), those from households with monthly income <200,000 UGX (aIRR = 1.798, CI = 1.306–2.477, P < 0.001), those delivered by cesarean section (aIRR = 1.835, CI = 1.291–2.610, P = 0.001), and those with **premature rupture of membranes (PROM) (**aIRR = 1.538, CI = 1.109–2.132, P = 0.010). The risk of hyperbilirubinemia requiring phototherapy was increased by over two times for the preterm (aIR = 2.776, CI = 1.978–3.895, P < 0.001), those in whom feeding was initiated after 1 hour (aIRR = 2.145, CI = 1.411–3.261, P < 0.001), instrumental delivery (aIRR = 2.005, CI = 1.109–3.625, P = 0.021) and ABO incompatibility (aIRR = 2.985, CI = 2.060–4.325, P < 0.001).

**Conclusion:**

2 in 10 neonates developed hyperbilirubinemia requiring phototherapy. The condition was associated with both modifiable factors, such as delayed initiation of breastfeeding, and non-modifiable factors, including ABO incompatibility, prematurity, and mode of delivery.

## 1. Background

Neonatal hyperbilirubinemia remains a significant global health concern, particularly in low- and middle-income countries. According to the Global Burden of Disease 2016 report, neonatal jaundice contributes to approximately 1,008 deaths per 100,000 live births, with the highest burden observed in South Asia and sub-Saharan Africa, where an estimated 1.1 million infants are affected annually ^1^ [1]. Clinically, neonatal hyperbilirubinemia is characterized by elevated serum bilirubin levels exceeding 85 µmol/L (5 mg/dL), although some studies define significant hyperbilirubinemia as total serum bilirubin levels exceeding 342 µmol/L in term and late preterm infants [2], typically presenting as jaundice, and a yellow discoloration of the skin, conjunctival icterus, and mucous membranes resulting from bilirubin deposition [3].

The pathophysiology of neonatal hyperbilirubinemia is multifactorial and involves **increased bilirubin production from the breakdown of fetal erythrocytes, impaired hepatic conjugation due to immature enzyme systems, and enhanced enterohepatic circulation** [4,5].

A wide array of maternal, neonatal, and genetic factors influences the risk and severity of neonatal hyperbilirubinemia. Preterm neonates are particularly susceptible because of underdeveloped hepatic function, which compromises bilirubin metabolism [5]. Hemolytic conditions such as Rh and ABO blood group incompatibilities contribute to elevated bilirubin levels through immune-mediated hemolysis [4]. Feeding practices also play a critical role; inadequate breastfeeding can result in dehydration and increased enterohepatic circulation, leading to early-onset breastfeeding jaundice within the first week of life [6]. Furthermore, genetic predispositions, such as polymorphisms affecting bilirubin conjugation, have been linked to higher rates of hyperbilirubinemia in East Asian and Mediterranean populations [7]. Additional risk factors include maternal infections of glucose-6-phosphate dehydrogenase (G6PD), gestational diabetes, and delayed meconium passage, all of which can contribute to elevated bilirubin levels [8].

Our study design therefore captures both early- and late-onset cases, providing a more comprehensive estimate of incidence. By determining the incidence and identifying key maternal and neonatal predictors of neonatal hyperbilirubinemia, this study contrasts with previous studies conducted in urban, better-resourced settings in Uganda and provides a clearer picture of the burden of hyperbilirubinemia at the regional referral hospital level. A study at Nsambya hospital showed the prevalence of neonatal hyperbilirubinemia was at 7.75% [9] and another conducted at Kawempe-Mulago hospital the prevalence was at 13.6% [10]. These studies were conducted in Kampala; Uganda’s capital and there’s no similar study conducted outside Kampala. The study conducted at Nsambya was retrospective and relied on chart reviews, which may have underestimated prevalence due to missing or incomplete data. The Mulago study focused exclusively on well neonates due for discharge between 24–72 hours of life, potentially missing cases of delayed-onset hyperbilirubinemia.

Findings from this study contribute to evidence-based screening and management strategies, particularly in resource-constrained settings where timely diagnosis and treatment remain a challenge.

## 2. Methods and materials

### 2.1. Study design and setting

MRRH is a publicly funded facility serving an estimated population of 610,600. The hospital has a functional clinical laboratory, overseen by a qualified laboratory technologist, and is equipped to perform serum bilirubin testing. The Department of Obstetrics and Gynecology at MRRH conducts over 200 deliveries per month, including spontaneous vaginal and operative deliveries. The NICU, under the Department of Pediatrics and Child Health, has 20 beds and three phototherapy units. Neonatal care is provided by a multidisciplinary team comprising pediatricians, senior house officers, intern doctors, and neonatal nurses.

Hospital (MRRH), located in north-central Uganda. The study was carried out from November 2024 to February 2025 in the postnatal ward and the Neonatal Intensive Care Unit (NICU). MRRH is a publicly funded facility serving an estimated population of 610,600. The hospital features a well-established clinical laboratory, overseen by a qualified laboratory technologist, and is equipped to perform accurate serum bilirubin assessments.

The Department of Obstetrics and Gynecology at MRRH conducts over 200 deliveries per month, including both spontaneous vaginal deliveries and operative births. The NICU, part of the Department of Pediatrics and Child Health, is equipped with 20 beds and three phototherapy units. Neonatal care is delivered by a multidisciplinary team comprising pediatricians, senior house officers, intern doctors, and neonatal nurses.

### 2.2. Study population

Eligible participants were mother–infant pairs with neonates born at 26 weeks of gestation or later. Gestational age was determined using the last menstrual period (LMP); when the LMP was unknown or uncertain, the New Ballard Score was used to estimate gestational age. The study included both inborn and out-born neonates who received care at Mubende Regional Referral Hospital.

### 2.3. Sample size determination

The sample size was calculated using OpenEpi (https://www.openepi.com/SampleSize/SSCohort.htm), based on a prior study conducted at Kawempe-Mulago Hospital, Kampala, Uganda, which reported that delayed initiation of feeding (>1 hour after birth) increased the risk of hyperbilirubinemia (adjusted odds ratio 2.74) [10].

Assuming 80% power and a 95% confidence level, and using the Fleiss formula for cohort studies, the minimum required sample size was 486 mother–infant pairs. Allowing for an 11% non-response or loss to follow-up rate, the final sample size was set at 539 mother–infant pairs.

### 2.4. Study variables

#### 2.4.1. Primary-outcome.

The primary outcome was neonatal hyperbilirubinemia requiring phototherapy, defined according to the American Academy of Pediatrics (AAP) guidelines. The cut-offs for initiating phototherapy were determined based on either gestational age and postnatal age as shown below:

**Table:** 

Phototherapy Thresholds According to Gestational Age	
Gestational Age (Weeks)	Unconjugated Bilirubin Level (mg/dL)
<28	5-6
28 – < 30	6-8
30 – < 32	8-10
32 – < 34	10-12
34 – < 35	12-14
≥35	>12
**Phototherapy Thresholds for ≥35 Weeks, Based on Postnatal Age**	
Postnatal Age (Hours)	Unconjugated Bilirubin Level (mg/dL)
≤24	>12
25–48	>15
49–72	≥18
>72	≥20

#### 2.4.2 Exposure variables.

Maternal sociodemographic characteristics (age, education level, occupation, and household income) were collected using a structured questionnaire administered to mothers. Obstetric and clinical characteristics, including history of urinary tract infection, diabetes mellitus, hypertension, preeclampsia, premature rupture of membranes, antenatal care attendance, and duration of labor, were abstracted from antenatal cards and medical records. Diagnoses of hypertension, diabetes, and preeclampsia were based on documented clinical assessments by attending clinicians.

Neonatal characteristics, including sex, gestational age, birth weight, multiple gestation, mode of delivery, birth trauma, Apgar score at 5 minutes, and feeding practices, were obtained from delivery records and direct observation. Gestational age was primarily determined using LMP, with the New Ballard Score used when LMP was unknown or uncertain. Neonatal sepsis was diagnosed clinically using Integrated Management of Neonatal and Childhood Illness (IMNCI) criteria without laboratory confirmation. Maternal and neonatal blood group and Rhesus status were determined using direct slide agglutination testing.

### 2.5 Data collection procedures

The principal investigator and a trained research assistant collected data after obtaining written informed consent from each participant. A structured questionnaire collected sociodemographic, obstetric, and clinical information. Each neonate underwent a physical examination to assess for birth trauma, and anthropometric measurements (weight and length) were recorded while unclothed for accuracy. Neonatal weight and length were measured while unclothed using a digital infant scale and a standard infant-meter, respectively, calibrated daily to ensure accuracy.

Venous blood samples (3 mL) were collected from each neonate via sterile venipuncture for serum bilirubin analysis and ABO/Rh blood grouping. Blood was drawn using standard aseptic techniques: venipuncture sites were disinfected with 70% alcohol and allowed to dry before collection. Samples were placed in serum separator tubes (SST) for bilirubin testing and in red-top, Ethylenediaminetetraacetic acid (EDTA)-free tubes for blood grouping. All samples were stored in light-protected biohazard transport containers and delivered to the hospital’s central laboratory within one hour.

Neonates were followed from birth up to 7 days of life, with serum bilirubin measurements performed at two predefined time points: within the first 24 hours of life (Day 1) and at Day 7, in accordance with the hospital follow-up schedule, with additional measurements obtained as clinically indicated.

### 2.6 Laboratory analysis

Total bilirubin levels were measured using a direct endpoint enzymatic method with the HumaStar 200 chemistry analyzer (HUMAN Diagnostics, Wiesbaden, Germany), which was calibrated daily. ABO and Rh blood grouping were performed using the standard direct slide agglutination technique. All analyses were completed within 24 hours of collection to ensure the reliability of the results. Clinical management of neonatal hyperbilirubinemia, including initiation of phototherapy, followed standard serum bilirubin reference (SBR) protocols based on gestational age and postnatal age thresholds, in accordance with American Academy of Pediatrics (AAP) guidelines [2].

### 2.7 Data management and statistical analysis

Data was coded, cleaned, and entered into Microsoft Excel (Office 2021) and subsequently exported to SPSS version 26 for analysis. The incidence of neonatal hyperbilirubinemia requiring phototherapy was calculated as the number of neonates diagnosed per 1,000 live neonates, excluding those lost to follow-up. This was reported with corresponding 95% confidence intervals (CIs). Modified Poisson regression analysis was used to identify risk factors of neonatal hyperbilirubinemia requiring phototherapy. Variables with a p-value < 0.05 in the bivariable analysis were considered for multivariable analysis. In the multivariable, backward stepwise regression was used till the final model fit was achieved. Only the variables that contributed to the final model were included in the multivariable table. A p-value < 0.05 was considered statistically significant. Results were presented as crude and adjusted incidence rate ratios (cIRR and aIRR) with 95% CIs.

#### 2.7.1 Ethical considerations.

This study was conducted in accordance with relevant ethical guidelines and regulatory standards. Ethical approval was obtained from the Kampala International University Institutional Research and Ethics Committee (IREC No: KIU-2024–515). The study adhered to national COVID-19 and Ebola safety protocols to ensure the well-being of both participants and research staff. Participant autonomy, privacy, and dignity were upheld throughout the study.

## 3 Results

### 3.1 Baseline characteristics of the study participants

A total of 539 mother–infant pairs were enrolled in this study. Of these, nearly two-thirds (60.3%) of the neonates were female, and less than one-third (27.3%) were preterm. The majority were exclusively breastfed (79.0%), while 64.4% initiated feeding within the first hour of life. An Apgar score below seven at 5 minutes was observed in 29.7% of neonates. ABO incompatibility was identified in 16.9%. The baseline characteristics of the study participants are **presented in** Table 1.

**Table 1:** Baseline maternal and neonatal characteristics of study participants (N = 539).

Characteristic	Frequency (n)	Percentage (%)
**Sociodemographic characteristics**		
Age of mother (years)		
<20	37	6.9
20–29	219	40.6
30–35	228	42.3
>35	55	10.2
Education		
Formal	253	46.9
Non-formal	286	53.1
Occupation		
Formal employment	122	22.6
Self-employed	338	62.7
Unemployed	79	14.7
Monthly income (UGX)		
<200,000	138	25.6
≥200,000	401	74.4
**Maternal clinical and obstetric characteristics**		
BMI		
Normal	427	79.2
Underweight	53	9.8
Overweight	42	7.8
Obesity	17	3.2
Hypertension		
No	515	95.5
Yes	24	4.5
Diabetes		
No	509	94.4
Yes	30	5.6
ANC visits		
1–3	148	27.5
4–6	361	67.0
>6	30	5.5
Preeclampsia		
No	504	93.5
Yes	35	6.5
PROM		
No	490	90.9
Yes	49	9.1
ABO blood group		
O	191	35.4
AB	64	11.9
B	174	32.3
A	110	20.4
Rhesus status		
Rh+	473	87.8
Rh−	66	12.2
**Neonatal characteristics**		
Sex		
Male	214	39.7
Female	325	60.3
Gestational age		
<37 weeks	147	27.3
≥37 weeks	392	72.7
Birth weight (g)		
<2500	202	37.5
≥2500	337	62.5
Feeding method		
Exclusive BF	426	79.0
Formula	40	7.4
Mixed	73	13.6
Feeding initiation		
<1 hour	347	64.4
>1 hour	192	35.6
Mode of delivery		
SVD	404	75.0
CS	123	22.8
Instrumental	12	2.2
APGAR score at 5 min		
<7	160	29.7
≥7	379	70.3
Birth trauma		
No	524	97.2
Yes	15	2.8
ABO incompatibility		
No	448	83.1
Yes	91	16.9
Rhesus incompatibility		
No	478	88.7
Yes	61	11.3
Neonatal sepsis		
No	499	92.6
Yes	40	7.4

**Abbreviations:** ANC, antenatal care; BMI, body mass index; BF, breastfeeding; SVD, spontaneous vaginal delivery; CS, cesarean section; PROM, premature rupture of membranes; Rh, Rhesus factor; APGAR, Appearance, Pulse, Grimace, Activity, Respiration.

### 3.2. Incidence of hyperbilirubinemia requiring phototherapy among neonates at Mubende Regional Referral Hospital

**Fig 1:**
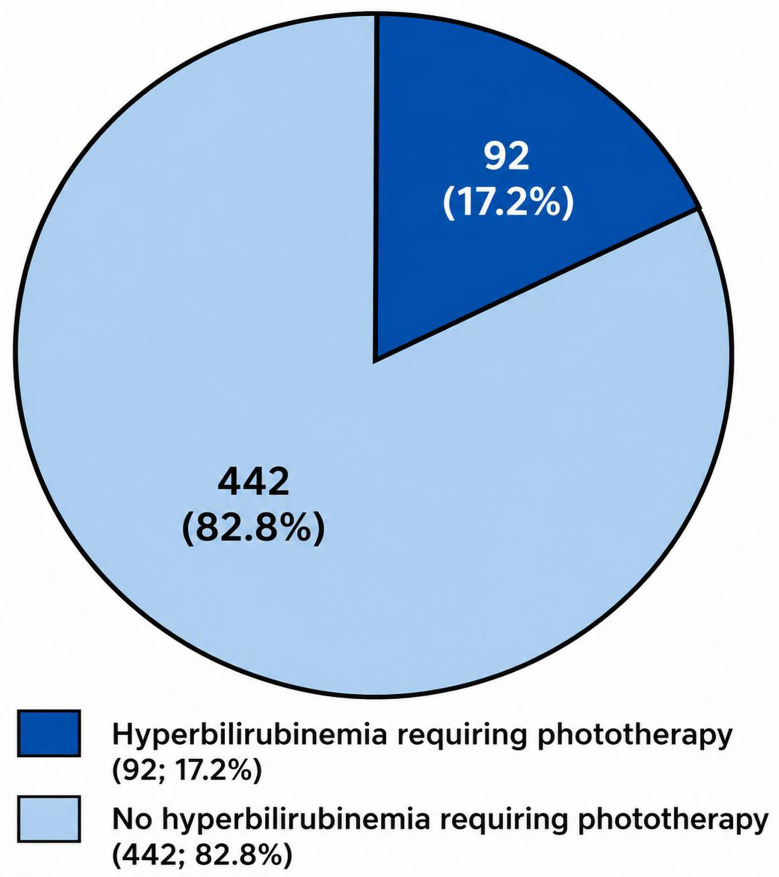
Proportion of neonates with and without hyperbilirubinemia requiring phototherapy among neonates enrolled at Mubende Regional Referral Hospital, Uganda (N  = 534).

Of the 539 mother–infant pairs enrolled, five neonates were lost to follow-up after discharge; therefore, 534 neonates completed follow-up. Overall, 92 of the 534 neonates developed neonatal hyperbilirubinemia requiring phototherapy, giving an incidence of 172 per 1,000 neonates Tables 2 and 3 (17.2%; 95% CI: 14.0–20.6) Fig 1.

**Table 2:** Bivariable analysis of risk factors for neonatal hyperbilirubinemia requiring phototherapy at Mubende Regional Referral Hospital.

Characteristic	No hyperbilirubinemia (N = 442)	Hyperbilirubinemia (N = 92)	cIRR	95% CI	P value
**Sociodemographic characteristics**					
Age of mother (years)					
<20	19 (4.3)	18 (19.6)	0.796	0.537–1.180	0.256
20–29	195 (44.1)	22 (23.9)	0.166	0.106–0.260	<0.001
30–35	207 (46.8)	19 (20.7)	0.138	0.085–0.222	<0.001
>35	21 (4.8)	33 (35.9)	1.00 (Ref)	–	–
Education					
Formal	217 (49.1)	32 (34.8)	1.00 (Ref)	–	–
Non-formal	225 (50.9)	60 (65.2)	1.638	1.105–2.429	0.014
Occupation					
Formal employment	102 (23.1)	19 (20.7)	1.00 (Ref)	–	–
Self-employed	295 (66.7)	40 (43.5)	0.760	0.459–1.260	0.288
Unemployed	45 (10.2)	33 (35.9)	2.694	1.655–4.387	<0.001
Monthly income (UGX)					
≥200,000	362 (81.9)	37 (40.2)	1.00 (Ref)	–	–
<200,000	80 (18.1)	55 (59.8)	4.393	3.040–6.349	<0.001
**Maternal clinical and obstetric characteristics**					
BMI					
Normal	387 (87.6)	36 (39.1)	1.00 (Ref)	–	–
Underweight	29 (6.6)	24 (26.1)	5.321	3.460–8.182	<0.001
Overweight	20 (4.5)	21 (22.8)	6.018	3.906–9.273	<0.001
Obesity	6 (1.4)	11 (12.0)	7.603	4.752–12.164	<0.001
Hypertension					
No	430 (97.3)	80 (87.0)	1.00 (Ref)	–	–
Yes	12 (2.7)	12 (13.0)	3.188	2.037–4.988	<0.001
Diabetes					
No	423 (95.7)	81 (88.0)	1.00 (Ref)	–	–
Yes	19 (4.3)	11 (12.0)	2.281	1.369–3.803	0.002
ANC visits					
>6	28 (6.3)	2 (2.2)	1.00 (Ref)	–	–
4–6	324 (73.3)	32 (34.8)	1.348	0.340–5.355	0.671
1–3	90 (20.4)	58 (63.0)	5.878	1.518–22.763	0.010
Preeclampsia					
No	419 (94.8)	81 (88.0)	1.00 (Ref)	–	–
Yes	23 (5.2)	11 (12.0)	1.997	1.181–3.377	0.010
Premature rupture of membranes (PROM)					
No	422 (95.5)	64 (69.6)	1.00 (Ref)	–	–
Yes	20 (4.5)	28 (30.4)	4.430	3.183–6.165	<0.001
**Neonatal characteristics**					
Sex					
Female	285 (64.5)	36 (39.1)	1.00 (Ref)	–	–
Male	157 (35.5)	56 (60.9)	2.344	1.601–3.432	<0.001
Gestational age					
≥37 weeks	353 (79.9)	35 (38.0)	1.00 (Ref)	–	–
<37 weeks	89 (20.1)	57 (62.0)	4.328	2.973–6.300	<0.001
Feeding initiation					
<1 hour	323 (73.1)	22 (23.9)	1.00 (Ref)	–	–
>1 hour	119 (26.9)	70 (76.1)	5.808	3.722–9.064	<0.001
Mode of delivery					
SVD	371 (83.9)	31 (33.7)	1.00 (Ref)	–	–
CS	65 (14.7)	55 (59.8)	5.944	4.024–8.780	<0.001
Instrumental	6 (1.4)	6 (6.5)	6.484	3.354–12.534	<0.001
Birth weight (g)					
≥2500	310 (70.1)	24 (26.1)	1.00 (Ref)	–	–
<2500	132 (29.9)	68 (73.9)	4.732	3.075–7.282	<0.001
APGAR score at 5 min					
<7	97 (21.9)	61 (66.3)	1.00 (Ref)	–	–
≥7	345 (78.1)	31 (33.7)	4.683	3.169–6.919	<0.001
ABO blood group incompatibility					
No	410 (92.8)	33 (35.9)	1.00 (Ref)	–	–
Yes	32 (7.2)	59 (64.1)	8.704	6.064–12.493	<0.001

**Abbreviations:** cIRR, crude incidence rate ratio; CI, confidence interval; BF, breastfeeding; SVD, spontaneous vaginal delivery; CS, cesarean section; ANC, antenatal care; PROM, premature rupture of membranes; LBW, low birth weight; Apgar, Appearance, Pulse, Grimace, Activity, and Respiration score; Rh, Rhesus factor.

**Table 3:** Multivariable analysis of risk factors for neonatal hyperbilirubinemia requiring phototherapy at Mubende Regional Referral Hospital.

Characteristic	Bivariable analysis			Multivariable analysis		
	cIRR	95% CI	P value	aIRR	95% CI	P value
**BMI of mother**						
Normal	1					
Underweight	5.321	3.460-8.182	<0.001	1.703	1.082-2.680	0.021
Overweight	6.018	3.906-9.273	<0.001	1.916	1.447-3.183	<0.001
Obesity	7.603	4.752-12.164	<0.001	1.930	1.233-3.022	0.004
**Income-monthly (ugx)**						
<200,000	4.393	3.040-6.349	<0.001	1.798	1.306-2.477	<0.001
>200,000	1					
**Gestational age in weeks**						
<37 + 6 days	4.328	2.973-6.300	<0.001	2.776	1.978-3.895	<0.001
≥37+	1					
**Feeding initiation**						
< 1 hr	1					
> 1 hr	5.808	3.722-9.064	<0.001	2.145	1.411-3.261	<0.001
**Mode of delivery**						
SVD	1					
CS	5.944	4.024-8.780	<0.001	1.835	1.291-2.610	0.001
Instrumental	6.484	3.354-12.534	<0.001	2.005	1.109-3.625	0.021
**PROM**						
No	1					
Yes	4.430	3.183-6.165	<0.001	1.538	1.109-2.132	0.010
**ABO incompatibility**						
No	1					
Yes	8.704	6.064-12.493	<0.001	2.985	2.060-4.325	<0.001

aIRR = adjusted incidence rate ratio; CI = confidence interval; BMI = body mass index; SVD = spontaneous vaginal delivery; PROM = premature rupture of membranes.

Following the back ward stepwise regression of the variables that were considered for multivariable analysis, only BMI, monthly income, gestational age, time of feeding initiation, mode of delivery, PROM and ABO incompatibility contributed to the final model and hence presented in the multivariable table (Table 3).

The risk of hyperbilirubinemia requiring phototherapy was increased by close to two times among neonates born to mothers who were underweight, overweight, or obese (based on maternal BMI during pregnancy) (aIRR = 1.703, CI = 1.082–2.680, P = 0.021; aIRR = 1.916, CI = 1.447–3.183, P < 0.001; aIRR = 1.930, CI = 1.233–3.022, P = 0.004), those from households with monthly income <200,000 UGX (aIRR = 1.798, CI = 1.306–2.477, P < 0.001), those delivered by cesarean section (aIRR = 1.835, CI = 1.291–2.610, P = 0.001), and those with premature rupture of membranes (aIRR = 1.538, CI = 1.109–2.132, P = 0.010). The risk of hyperbilirubinemia requiring phototherapy was increased by over two times for the preterm (aIR = 2.776, CI = 1.978–3.895, P < 0.001), those in whom feeding was initiated after 1 hour (aIRR = 2.145, CI = 1.411–3.261, P < 0.001), instrumental delivery (aIRR = 2.005, CI = 1.109–3.625, P = 0.021) and ABO incompatibility (aIRR = 2.985, CI = 2.060–4.325, P < 0.001).

## 4. Discussion

This study determined the incidence and predictors of neonatal hyperbilirubinemia requiring phototherapy at a tertiary healthcare facility in Uganda. Among the 534 neonates who completed follow-up, 92 developed neonatal hyperbilirubinemia requiring phototherapy, giving an incidence of 172 per 1,000 neonates (17.2%; 95% CI: 14.0–20.6%). This demonstrates a substantial burden of neonatal hyperbilirubinemia in this setting.

The observed incidence is lower than that reported at Nsambya Hospital, Kampala, where 227 per 1,000 neonates (22.7%) required phototherapy [9], but higher than the incidence reported at Mulago National Referral Hospital, Kampala, of 136 per 1,000 neonates (13.6%) [10]. These differences are likely due to variations in study design, timing of bilirubin measurement, and study populations. The Mulago study assessed bilirubin only at discharge between 24 and 72 hours of life, which may have missed cases of delayed-onset hyperbilirubinemia [10]. Differences in inclusion criteria and health-care infrastructure may also have influenced the reported incidences. These findings indicate that neonatal hyperbilirubinemia remains a significant clinical and public health concern in Uganda. The variation in incidence between facilities emphasizes the need for standardized monitoring protocols, early identification of at-risk neonates, and timely interventions to reduce morbidity associated with hyperbilirubinemia [10,11].

Studies from other low- and middle-income countries have reported variable incidences of neonatal hyperbilirubinemia. In Ethiopia, incidences of 205 and 246 cases per 1,000 neonates have been reported, while in Rwanda an incidence of 443 cases per 1,000 neonates was observed [11,12,13]. Most of these studies included neonates admitted to neonatal intensive care units, who are more likely to develop hyperbilirubinemia because of comorbid illnesses and complications.

In Cameroon, a lower incidence of 152 cases per 1,000 neonates was reported [14], which is lower than in our study. However, that study used retrospective data, which may have missed some cases. In Nigeria, a much higher incidence of 402 cases per 1,000 neonates was reported [15], likely because the study included hospitalized newborns who were more vulnerable. Similarly, a study from Iraq reported an incidence of 350 cases per 1,000 neonates [16], but this also involved hospitalized neonates, many of whom had other medical conditions. Differences in study design, inclusion criteria, and health-care settings likely explain the wide variation in reported incidences.

In this study, multivariable analysis identified seven independent predictors of neonatal hyperbilirubinemia requiring phototherapy: abnormal maternal BMI, low household income, prematurity, delayed initiation of feeding, cesarean or instrumental delivery, premature rupture of membranes, and ABO incompatibility.

Abnormal maternal BMI was significantly associated with increased risk of neonatal hyperbilirubinemia. This finding suggests that both maternal undernutrition and overnutrition may adversely influence neonatal bilirubin metabolism and early neonatal adaptation. Similar associations have been reported in studies from Ethiopia [11,12]. Maternal undernutrition has been linked to placental insufficiency and preterm birth, which predispose neonates to immature hepatic enzyme systems and reduced bilirubin conjugation [11]. In contrast, maternal overnutrition is associated with metabolic and inflammatory changes that may impair fetal liver maturation and bilirubin metabolism [12].

Low household income status was also associated with an increased risk of neonatal hyperbilirubinemia requiring phototherapy. Neonates born to mothers from low-income households were nearly twice as likely to develop hyperbilirubinemia compared with those from higher-income households, consistent with findings from Nigeria [15]. Low household income is often associated with inadequate maternal nutrition, limited access to quality antenatal care, and delayed health-seeking behavior, all of which increase the risk of adverse perinatal outcomes. Financial constraints may also limit access to early postnatal follow-up and delay the recognition and management of neonatal jaundice, allowing bilirubin levels to rise to levels requiring phototherapy [15].

Neonates delivered by cesarean section were nearly twice as likely to have hyperbilirubinemia compared with those born via spontaneous vaginal delivery. A study conducted in Ethiopia and Uganda showed similar associations [10,11]. Cesarean delivery is often associated with delayed initiation of breastfeeding due to postoperative maternal pain, sedation, and separation of the mother–infant dyad. Inadequate early feeding may lead to dehydration and increased enterohepatic circulation of bilirubin, resulting in elevated serum bilirubin levels. Additionally, neonates born by cesarean section may experience delayed physiological adaptation and altered hepatic enzyme activity, which can further impair bilirubin conjugation and clearance. In emergency cesarean sections, underlying obstetric complications such as fetal distress or prolonged labor may also contribute to increased bilirubin production [10,11].

Premature rupture of membranes was significantly associated with an increased risk of neonatal hyperbilirubinemia requiring phototherapy. Similar associations have been reported in Rwanda [13] and Ethiopia [11]. This finding suggests that intrapartum and perinatal infectious and inflammatory processes contribute to the development of neonatal hyperbilirubinemia. PROM predisposes neonates to early-onset sepsis, which can impair hepatic bilirubin conjugation through inflammation-mediated cholestasis and reduced hepatocellular function. In addition, subclinical infections and systemic inflammation may increase hemolysis and bilirubin production, further elevating serum bilirubin levels. Neonates born after PROM are also more likely to experience delayed initiation of breastfeeding or require closer clinical observation, which may indirectly contribute to bilirubin accumulation [11,17].

Prematurity was a strong independent predictor of neonatal hyperbilirubinemia requiring phototherapy, with preterm neonates being nearly three times more likely to develop clinically significant hyperbilirubinemia compared with term neonates. Similar findings were reported at Mulago National Referral Hospital in Kampala [10]. Preterm infants have immature hepatic enzyme systems, particularly reduced activity of uridine diphosphate-glucuronosyltransferase (UGT1A1), which limits bilirubin conjugation and excretion [10,11].

This study found that neonates whose mothers-initiated breastfeeding after the first hour of birth were more than twice as likely to develop neonatal hyperbilirubinemia requiring phototherapy, consistent with findings from Ethiopia [11]. Early breastfeeding promotes the passage of meconium, which facilitates bilirubin excretion and reduces its accumulation [11]. This finding emphasizes the importance of promoting immediate or early initiation of breastfeeding within the first hour of life as a practical and cost-effective intervention to prevent severe neonatal hyperbilirubinemia.

ABO incompatibility showed the strongest association in this study, with nearly a threefold increased risk of neonatal hyperbilirubinemia requiring phototherapy. This condition, particularly when mothers with blood group O develop antibodies against a neonate with A or B blood group, leads to hemolysis and subsequent hyperbilirubinemia. Our findings are consistent with data from Turkey, Egypt, Nepal, India, Nigeria, and Pakistan [18–20]. This association was also confirmed in a previous study conducted at Mulago [10].

## 5. Strengths and limitations

The strengths of this study include its prospective cohort design, which minimized recall bias, and the use of total serum bilirubin measurement, which improved diagnostic accuracy. The relatively large sample size and inclusion of both early- and late-onset cases also strengthened the reliability of the incidence estimate.

However, some limitations should be considered. Glucose-6-Phosphate Dehydrogenase (G6PD) deficiency, an important contributor to neonatal hyperbilirubinemia, was not assessed, which may have led to underestimation of the true burden of the condition in this setting where Glucose-6-Phosphate Dehydrogenase (G6PD) deficiency, is common. Neonatal sepsis was diagnosed using Integrated Management of Neonatal and Childhood Illnesses (IMNCI) clinical criteria without laboratory confirmation such as C-Reactive Protein (CRP) or blood cultures, which may have resulted in misclassification. Gestational age estimation based on the last menstrual period may have been affected by recall bias, particularly among women with irregular menstrual cycles or early pregnancy bleeding. Furthermore, neonatal assessments were conducted at discrete time points (Day 1 and Day 7) rather than through daily bilirubin monitoring, which may have limited the precision in capturing the timing and incidence of hyperbilirubinemia.

## 6. Conclusion

This study found a moderately high incidence of neonatal hyperbilirubinemia, affecting almost 2 in 10 neonates in a tertiary hospital in Uganda. Abnormal maternal BMI, low household income, prematurity, delayed initiation of feeding, cesarean or instrumental delivery, premature rupture of membranes, and ABO incompatibility were significant independent predictors of hyperbilirubinemia.

Routine screening for hyperbilirubinemia within health-care facilities, especially during the first week of life, should be strengthened. Screening for ABO incompatibility should be integrated into routine maternal and neonatal care. Public health efforts should emphasize early initiation of breastfeeding, maternal nutritional support, and caregiver education on early signs of hyperbilirubinemia and timely care-seeking. Expanding access to phototherapy in peripheral health facilities and strengthening referral systems will be critical in reducing delays in treatment and improving neonatal outcomes.

## Supporting information

S1 File Hyperbilubinemia Hamdi Data edited relevant. (SAV)
